# *LncRNA CRNDE* facilitates epigenetic suppression of *CELF2* and *LATS2* to promote proliferation, migration and chemoresistance in hepatocellular carcinoma

**DOI:** 10.1038/s41419-020-02853-8

**Published:** 2020-08-11

**Authors:** Shu-Cai Xie, Jian-Quan Zhang, Xi-Li Jiang, Yong-Yong Hua, Shao-Wei Xie, Ye-Ang Qin, Yi-Jun Yang

**Affiliations:** 1 Department of Hepatobiliary Surgery, Haikou People’s Hospital /Affiliated Haikou Hospital of Xiangya Medical College, Central South University, Haikou, 570208 Hainan Province People’s Republic of China; 2 Department of Radiology, The Second People’s Hospital of Hunan Province/Brain Hospital of Hunan Province, Changsha, 410007 Hunan Province People’s Republic of China

**Keywords:** Tumour angiogenesis, Cancer

## Abstract

Our study aimed to investigate the expression, functional significance, and related mechanism of long noncoding RNA CRNDE (colorectal neoplasia differentially expressed) in hepatocellular carcinoma (HCC) pathogenesis. The resulted revealed that CRNDE was significantly overexpressed in HCC tissues and cell lines, and was statistically correlated with poor clinical outcome. CRNDE knockdown markedly decreased HCC cell proliferation, migration, and chemoresistance. In addition, in vivo experiments confirmed the suppressive effect of CRNDE knockdown on HCC progression. Mechanically, CRNDE directly bound to EZH2 (enhancer of zeste homolog), SUZ12 (suppressor of zeste 12), SUV39H1, and mediated their inhibition of tumor suppressor genes, including CUGBP Elav-like family member 2 (CELF2) and large tumor suppressor 2 (LATS2). CELF2 exerted tumor suppressive effect in HCC and was involved in CRNDE-mediated oncogenic effect. In addition, the oncogenic effects of CRNDE on HCC proliferation, migration and tumorigenesis, as well as its inhibition of Hippo pathway were abolished by LATS2 overexpression. Together, our work demonstrated the importance of CRNDE in HCC progression and elucidated the underlying molecular mechanisms. These findings provided new insights into HCC pathogenesis and chemoresistance mediated by CRNDE.

## Introduction

Hepatocellular carcinoma (HCC), which is a type of hepatoma, is the most common primary cancer in liver and the third leading cause of cancer mortality worldwide^1^. HCC can be caused by chronic hepatitis B virus and hepatitis C virus infections, cirrhosis, heavy alcohol drinking, or diabetes^2^. Numerous clinical approaches for HCC treatment exist, including surgical resection, liver transplantation, and medical treatment, such as radiation therapy, transarterial chemoembolization, and molecular targeted chemotherapy^3,4^. However, HCC is a heterogeneous and complex tumor in which multiple molecular signaling pathways are dysregulated, the therapeutic benefits of the currently available treatments are often reduced by their limited efficacy^5^, failure of early diagnosis^6^, and acquired drug resistance^7^. Thus, improving our knowledge of HCC pathophysiology at molecular levels may maximize clinical effectiveness in the future HCC treatment.

Long noncoding RNAs (lncRNAs) are a type of RNA that usually contain >200 nucleotides. In recent years, growing evidence indicates that lncRNAs are implicated in diverse human diseases, including autoimmune disease^8^, cardiovascular disease^9^, neurodegenerative disease^10^, and different cancer types^11–14^. Several lncRNAs were reported to promote HCC progression by antagonizing *let-7*, interacting with *CTNNB1*, or targeting miR-26a-5p/GSK3β and Wnt/β signaling pathways^15–17^. The present study focused on lncRNA CRNDE (colorectal neoplasia differentially expressed), which has been demonstrated previously as an oncogene in colorectal cancer^18^, bladder cancer^19^, glioma^20^, and lung cancer^21^. Although CRNDE was widely reported to be upregulated in HCC and promote HCC development via multiple miRNAs, including miR-136, miR-126, miR-337, miR-384, and miR-203 (refs. ^22–26^). However, whether CRNDE facilitated chemoresistance of HCC had not been reported. Thus, in present study, we aimed to investigate the detailed mechanisms of CRNDE in HCC chemoresistance and pathogenesis.

Polycomb repressive complexes (PRC) are formed by polycomb-group proteins and classified in two major groups: PRC1 and PRC2 (ref. ^27^). We focused in the present study on PRC2, which has histone methyltransferase activity and consists of the catalytic subunit enhancer of zeste homolog (EZH1 or EZH2), suppressor of zeste 12 (SUZ12), embryonic ectoderm development (EED)^28^. Dysregulated expression and function of PRC2 were observed in diverse human cancers, and accumulating evidence indicated that PRC2 played important tumor suppressive or oncogenic roles by epigenetically modulating target gene expression in tumorigenic processes^29,30^. For instance, EZH2, the key subunit of PRC2 was found aberrantly expressed in HCC patients, and EZH2 overexpression was closely associated with HCC progression^31,32^. Furthermore, EZH2 knockdown impaired HCC tumorigenesis by reducing histone H3 lysine 27 methylation levels. Interestingly, the transcriptional repressive function of H3K27me3 could be also regulated by the histone-lysine *N*-methyltransferase SUV39H1 (refs. ^33,34^), which was reported to exert tumor-promoting function by modulating H3K27me3 modification^34,35^. Many studies revealed that PRC2 regulated the epigenetic state in a cancer cell by directly interacting with different nuclear lncRNAs and influencing the histone marks deposition, DNA methylation or chromosomal architecture^36,37^. It has been demonstrated that lncRNA CRNDE promoted the colorectal cancer progression by binding to, EZH2 (ref. ^18^). Despite the critical function of PRC2 for the balance of cancer cell proliferation and differentiation in tumorigenesis, global genes involved in PRC2 regulation during HCC progression and the underlying molecular mechanisms remain to be elucidated.

In the present study, we showed that lncRNA CRNDE was significantly upregulated in HCC tissues and silencing of CRNDE inhibited HCC cell proliferation, migration, and chemoresistance. Given the importance of PRC2 as a histone methyltransferase in HCC, we further investigated the interaction between CRNDE and EZH2, SUZ12, and SUV39H1, and found that the inhibitory effect of EZH2, SUZ12, and SUV39H1 on tumor suppressors was mediated by CRNDE. In addition, we provided the first evidence that CUGBP Elav-like family member 2 (CELF2), an RNA-binding protein, was downregulated in HCC patients and cell lines, and its overexpression suppressed the tumorigenesis, migration, and chemoresistance in HCC. Furthermore, we discovered that the inhibition effect of CRNDE knockdown on HCC proliferation and migration was compromised by the silencing of CELF2, whereas overexpression of human large tumor suppressor 2 (LATS2), the Hippo signal pathway kinase, could efficiently inhibit the oncogenic properties of CRNDE in HCC.

## Materials and methods

### Clinical specimens and ethic statement

Forty seven pairs of fresh HCC tumor tissues and the matched adjacent nonmalignant tissues were collected with written informed consent from enrolled HCC patients underwent surgery, and did not received chemoradiotherapy prior to surgery at Haikou People’s Hospital/Affiliated Haikou Hospital of Xiangya Medical College from 2016 to 2018. The study was approved by the Ethics Committee of Haikou People’s Hospital/Affiliated Haikou Hospital of Xiangya Medical College, Central South University and all procedures were performed in accordance with relevant regulations. Patients information is summarized in Table 1.

**Table 1:** Clinical pathological characteristics of 47 HCC patients.

Clinicopathologic	
parameters	Number
Frequency	
Gender	
Male	31
Female	16
Age (years)	
≤60	36
>60	11
Hbsag	
Negative	29
Positive	18
Cirrhosis	
Absence	13
Presence	34
AFP	
≤20	23
>20	24
TNM	
T1/T2	20
T3/T4	27
Microvacular invasion	
Absence	15
Presence	32
Macrovacular invasion	
Absence	20
Presence	27
Encapsulation	
Absence	14
Presence	33

### Cell culture

Human hepatoma cell lines HCCC-9810, Bel-100, Bel-7405, Bel-7402, Huh-7, WRL68, SMMC-7721, HepG2, and the immortalized human hepatic cell line THLE3 were purchased from the Type Culture Collection of the Chinese Academy of Sciences (Shanghai, China). HCCC-9810, Bel-7402, Bel-100, Bel-7405, and SMMC-7721 were cultured in RPMI media 1640 (Gibco, USA), while Huh-7, WRL68, and HepG2 were cultured in DMEM supplemented with 10% fetal bovine serum (FBS; Gibco, USA), 100 U/ml penicillin, and 0.1 mg/ml streptomycin (Sigma, USA). Multidrug-resistant Huh-7/ADR (adriamycin) cell line was purchased from Fenghui Biotechnology (Changsha, Hunan) and cultured in the above-described complete RPMI media 1640 with 1 mg/L of ADR. The cells were maintained under standard culture conditions (37 °C, 5% CO_2_). Cells were validated by STR and tested routinely to make sure cell without mycoplasma contamination.

### IHC staining

HCC animal tumor tissues were fixed in 10% formalin solution, embedded in paraffin, and frozen at −80 °C. The tissues were cut into 4 mm thick sections using a cryostat and the sections were mounted onto gelatin-coated histological slides. The slides were deparaffinized, rehydrated, and blocked with 5% nonfat milk for at room temperature (RT) for 1 h. Primary antibody against Ki67 was applied to the tissue sections and incubated overnight at 4 °C. The next day, the slides were washed three times in wash buffer, followed by incubation with HRP-labeled secondary antibody for 1 h at RT. The slides were counterstained with hematoxylin and mounted with a glycerol antifade mounting media to visualize under microscope.

### H&E staining

Tissues were fixed with 10% paraformaldehyde for 24 h and embedded in paraffin wax. The samples were cut into coronal sections of 4 mm in thickness and deparaffinized by flaming the sections on burner, followed by two changes of xylene and rehydration with alcohol. The slides were stained with hematoxylin for 10 min, rinsed in running tap water for 10 min and differentiated with 0.3% acid alcohol. Next, the slides were counterstained with 0.5% eosin for 2 min, dehydrated with alcohol, and cleared with three changes of xylene till they were totally transparent. A few drops of Permount mounting medium was placed onto the slides and coverslips were gently added. The slides were dried overnight in the hood and observed under microscope.

### In vivo tumor formation and lung metastasis models

Animal experiments were performed as previously described with minor modifications^38^. The investigators were blinded to the group allocation during the experiment and when assessing the outcome. Four-week-old male BALB/C nude mice were purchased from Hunan Slac Jingda Laboratory Animal Co., Ltd (Hunan, China), and divided randomly into two groups (*n* = 8 per group, 16 total). A total of 200 µl of 10^7^ Bel-100 cells transfected with lentiviruses carrying shCRNDE or shNC were injected subcutaneously into each mouse at a single side of the posterior flank, and tumor formation was evaluated by measuring the tumor sizes every 5 days. Tumor sizes were calculated using the formula *V* = 0.5 × *D* × *d*^2^ (*V* is tumor volume, *D* is longitudinal diameter, and *d* is latitudinal diameter). Mice were sacrificed 30 days after the injection and tumor tissues were isolated for photography.

Sixteen BALB/C nude mice were randomly divided into two group. To develop the in vivo metastasis model, each of eight mice was injected with 1 × 10^7^ Bel-100 cells (transfected with shCRNDE or shNC) via the tail veins (*n* = 8 per group). Mice were sacrificed after 30 days and the lung tissues were isolated for photography. Pulmonary nodules were numerated, and pulmonary migration was analyzed using hematoxylin–eosin (H&E) staining. All animal study was approved by the Ethics Committee of Haikou People’s Hospital/Affiliated Haikou Hospital of Xiangya Medical College, Central South University and all procedures were performed in accordance with relevant regulations.

### Western blotting and antibodies

Cell were lysed in chilled RIPA lysis buffer containing protease inhibitor cocktail (Sigma, USA) and centrifuged at 4 °C for 15 min at 12,000 × *g*. A total of 60 μg of total protein from the cleared lysate was electrophoresed with SDS–PAGE and transferred to PVDF membrane (Merck Millipore), followed by 1 h incubation with 5% nonfat milk. β-actin was used as a loading control. The membranes were incubated with the corresponding primary antibodies against EZH2 (1:1000 dilutions, Cell Signaling Technology, #4905), BIK (1:1000 dilutions, Cell Signaling Technology, #4592), LATS2 (1:1000 dilutions, Cell Signaling Technology, #5888), p27KIP1 (1:1000 dilutions, Cell Signaling Technology, #3686), CELF2 (1:1000 dilutions, Abcam, ab111728), yes-associated protein (YAP; 1:1000 dilutions, Cell Signaling Technology, #14074), and p-YAP (1:1000 dilutions, Cell Signaling Technology, #13008) at 4 °C overnight. The membranes were washed extensively in 1× PBST and incubated with HRP secondary antibodies (1:3000 dilutions, Abcam, ab205718) at RT for 1 h. Protein bands were detected by applying ECL substrates (Pierce) and visualized under an Odyssey infrared scanner (Li-Cor Biosciences Inc).

### RNA isolation and qPCR analysis

Total RNA from the tissue samples and cell lines was isolated using TRIzol (Invitrogen). After assessing the RNA purity and concentration by UV spectrometry, first-strand cDNA was generated using 2 μg total RNA and TAKARA Reverse Transcription Kit (Takara, Dalian, China). Real-time qPCR was performed using the SYBR Green qPCR Kit (Takara, Dalian, China). GAPDH was used as an endogenous expression control for mRNA and lncRNA. The primers were synthesized by Shengong Ltd. Co. (Shanghai, China). All the sequences of the primers were listed in Table 2. TaqMan miRNA assays were performed for miRNA analysis according to the manufacturer’s protocol. The relative gene expression level was calculated using the comparative Ct method.

**Table 2:** Primers list.

GENE	Forward primer	Reverse primer
CRNDE	AAATTCATCCCAAGGCTGGT	AAACCACTCGAGCACTTTGA
CELF2	TCCACAGGAATTTGGAGACC	TTGGATAGCAGCTTGTGCAG
p15	GATCCCAACGGAGTCAACC	ACCAGCGTGTCCAGGAAG
p27KIP1	GCAAGTACGAGTGGCAAGAG	CCAAATGCGTGTCCTCAGAG
LATS2	ACAAGATGGGCTTCATCCAC	CTCCATGCTGTCCTGTCTGA
BIK	ACCATGGAGGTTCTTGGCAT	GCTCACGTCCATCTCGTCC
FAT4	AGTGTTTTGGCTACTGTCATTGCT	GCACTTCGGTGGGGTAGGT
KLF6	AGTTTACCTCCGACCCCATT	AAGGCTTTTCTCCTGGCTTC
GAPDH	CTGACTTCAACAGCGACACC	GTGGTCCAGGGGTCTTACTC

### Plasmid construction and cell transfection

Short hairpin RNAs targeting CRNDE and siRNA targeting CELF2, SUZ12, EZH2, SUV39H1, and the respective negative control shRNA was synthesized by GenePharma (Shanghai, China). Target sequence were shown in Table 3. The pLKO.1-shCRNDE plasmid was generated by subcloning shRNA CRNDE #2 into pLKO.1 vector. To generate lentivirus, HEK293T cells were co-transfected with pLKO.1-shCRNDE plasmid, psPAX2 packaging plasmid, and pMD2.G envelop plasmid using Lipofectamine 3000 (Invitrogen). HCC cells were cultured in six-well plate at a density of 5 × 10^4^ cells/ml and transduced with the titer-determined lentiviruses. Other constructed plasmids were transiently transfected into HCC cells.

**Table 3:** Sequence of shRNA or siRNA.

	**shRNA target sequence**
shRNA#1 CRNDE	GCCGTTGGTCTTTGAAATTTC
shRNA#2 CRNDE	GCTCGAGTGGTTTAAATATGT
shRNA#3 CRNDE	GATGTGTTTCAATCTAGATGC
	**siRNA sequence**
siRNA CELF2	GCAAACCUUACUGAUCCUA
SiRNA SUV39H1	ACCUCUUUGACCUGGACUA
siRNA EZH2	GAGGUUCAGACGAGCUGAUUU
siRNA SUZ12	CAUCGAAACUCCAGAACAA

The full-length CRNDE (NM_001308963.1), LATS2 (NM_014572.3), and CELF2(NM_006561.3) cDNA were synthesized by GenePharma and subcloned into the expression vector pcDNA3.1(+) (Invitrogen). The pcDNA3.1(+) plasmids were transiently transfected into HCC cells using Lipofectamine 3000 according to the user’s manual. Cells were harvested at 48 h post transfection and subjected to other indicated assays.

### Cell viability assay (MTT assay)

MTT assay was performed using MTT Cell Proliferation Assay Kit (Abcam, UK) according to the user’s guide. Briefly, the culture medium was discarded at 0, 24, 48, and 72 h after transfection, and new serum-free DMEM supplemented with MTT (3-(4, 5-dimethylthiazol-2-yl)−2, 5-diphenyltetrazoliumbromide) reagent. The cells were incubated at 37 °C for 3 h and MTT solvent was added. After shaking the mixture for 15 min, the absorbance at OD 490 was measured at EnSpire-Multimode Plate-Reader (PerkinElmer).

### Cellular colony formation assay

Bel-100 and Huh-7 cells were diluted in DMEM with 10% FBS single-cell suspensions were prepared. Cell numbers were carefully recorded and cells were plated in a six-well plate at a density of 100 cells per well. Upon the lentiviral infection with shCRNDE, shNC, CELF2, or empty vectors, cells were further incubated at 37 °C for 7 days, then fixed with 10% neutral buffered formalin and stained with 0.1% crystal (w/v) violet for 30 min. Colony formation was examined under a light microscope and the visible colonies that contained at least 50 individual cells were counted.

### Transwell assay

Cell migration and invasion was assessed by the transwell assay. Poly-carbonate transwell filters (8 µm, Corning) were inserted over the lower chambers that were filled with DMEM supplemented with 10% FBS. A total of 5 × 10^4^ cells were suspended in serum-free medium and seeded into the upper chambers. Cell invasion assay through extracellular matrix was conducted with slight modifications. Matrigel matrix (Corning) was thawed on ice, mixed with Tris-NaCl coating buffer, gently loaded on each permeable support, and solidified by incubating at 37 °C for 1 h. Cells were plated on top of the thin Matrigel coating layer and 15% FBS was added to each receiver well as chemoattractant. After incubation at 37 °C overnight, cells on the lower surface were fixed with 100% methanol and stained with 0.4% crystal violet for 30 min. The transmitting and invading cells were counted and photographed under optical microscope.

### RNA immunoprecipitation

Bel-100 and Huh-7 cells were harvested, and nuclei were pelleted by centrifugation at 2500 × *g* for 10 min. RNA immunoprecipitation assay (RIP) was performed using Magna RIP^TM^ RNA-Binding Protein Immunoprecipitation Kit (Millipore, MA, USA) according to the user guides. Briefly, nuclei were resuspended and lysed in ice cold RIP lysis buffer, followed by incubation with protein A Sepharose beads that were conjugated with antibodies against EZH2, SUZ12, SUV39H1, or the control rabbit immunoglobulin G (IgG; Santa Cruz Biotechnology, USA) at 4 °C for 10 h. The beads were washed thoroughly and the purified RNA was used for qRT-PCR analysis.

### RNA pull-down assay

Bel-100 cells were harvested by trypsinization, quenched in DMEM supplemented with 10% FBS, and resuspended in nuclear isolation buffer. Nuclei was pelleted by centrifuging at 700 × *g* for 15 min and resuspended in ice cold RIP buffer supplemented with protease inhibitors and DTT. CRNDE was cloned into the pcDNA3.1 vector under T7 promoter control and in vitro transcription was performed using 1 µg linearized plasmid, 2 µL T7 polymerase (20 U/µL, Promega), and 2 µL biotin RNA labeling mix (Roche). The biotinylated RNAs were treated with 2 µL RNase-free DNase I (Qiagen, Germany) purified with G50 Sephadex Quick Spin columns (Sigma, USA). The purity and concentration of the eluted RNA was controlled using a Nanodrop. The purified RNA was incubated with the nuclear extract at RT for 1 h, further followed by incubation with 60 µL Streptavidin agarose beads (Invitrogen) at RT for 1 h. Finally, the beads were washed extensively and incubated with protein loading buffer to elute RNA–protein complexes, followed by SDS–PAGE and western blot (WB) analysis with indicated targeted protein antibodies. In addition, HuR (Human antigen R) and AR RNA were used as positive control.

### Chromatin isolation by RNA purification

Chromatin isolation by RNA purification (ChIRP) experiment was performed as previously described with minor modifications^39^. Briefly, CRNDE-specific antisense DNA tilting probes were designed by using the online software singlemolecule.com. Cells transfected with shCRNDE or shNC were harvested and RNA–chromatin interactions were preserved by adding freshly prepared 1% glutaraldehyde (Sigma, USA). Cross-linking reaction was quenched by incubation with 1.25 M glycine at RT, and the cross-linked cells were lysed in lysis buffer supplemented with protease inhibitor cocktail (Sigma, USA) and RNAse inhibitor (Sigma, USA), followed by sonication in Bioruptor (Diagenode) at 4 °C till the lysate turned clear. Biotinylated probes were hybridized to RNA by shaking at 37 °C for 4 h, and bound chromatin was isolated using Invitrogen magnetic beads. RNA eluent was further analyzed by qPCR. LacZ was used as negative control.

### ChIP-qPCR

HCC cells were treated with 1% formaldehyde at RT for 10 min to form reversible DNA–protein links and 2 mL of 10× glycine was added to each dish to quench excess formaldehyde. The cells were then washed by cold PBS harvested and resuspended in 0.5 mL of nuclei isolation buffer containing 2.5 µL of 200× protease inhibitor cocktail III and spin the cell suspension at 800 × *g* at 4 °C for 5 min, followed by 30 min sonication. DNA concentration and fragment size were determined by UV spectrometry and agarose gel, respectively. The chromatin was diluted with RIPA buffer and immunoprecipitated with the primary antibodies against EZH2, SUZ12, SUV39H1, H3K27me3, H3K9me3 (Millipore, USA), or the control IgG at 4 °C for 1 h. Protein A/G beads were prepared and added for incubation at 4 °C overnight. Finally, the immunoprecipitated samples were washed with low salt and high salt wash buffer, and DNA was eluted for qRT-PCR analysis. The primers used for CHIP and CHIRP were listed in Table 4.

**Table 4:** The list of primers for CHIP and CHIRP.

GENE	Forward primer	Reverse primer
p27KIP1	AAGTGCCGCGTCTACTCCTG	TGGAGGCAGTGGGCAATGGT
LATS2	GGCAGGAGGATGGCTTGA	GCCCTACTGGCATTACC
BIK	CAAGCTTGCAGAACAGCAGG	TGGCATTGGCAACAGAACC
CELF2	GCCAAAAGCCAAATCCCACC	GGGTTCCGGGTGCTTTGATA

### Sphere formation

Bel-7405 cells were diluted in DMEM sphere formation medium supplemented with 100 U/ml penicillin, 0.1 mg/ml streptomycin, 20 ng/ml human epidermal growth factor (Sigma, USA), and 1% B27 supplement (Invitrogen, USA), and seeded in an ultra-low attachment six-well plate at a density of 5 × 10^3^ cells per well. After lentiviral infection with CRNDE, CRNDE+LATS2 or empty vector, cells were further incubated at 37 °C for 10 days. Sphere morphology was observed under a microscope every 2 days and total number of spheres >50 µm was recorded.

### Statistical analysis

All experiments have been conducted independently at least three times. Data were presented as mean ± standard deviation and analyzed by GraphPad Prism 6.0 software. Data meet normal distribution, and variance was similar between the groups that are being statistically compared. Student’s *t*-test was applied to evaluate the differences between two groups, while one-way ANOVA followed by Tukey’s post test was applied for multiple groups. A value of *P* < 0.05 was considered as significant difference for all analyses.

## Results

### CRNDE was overexpressed in both HCC patients and cell lines, and was positively correlated with poor prognosis

**Fig. 1:**
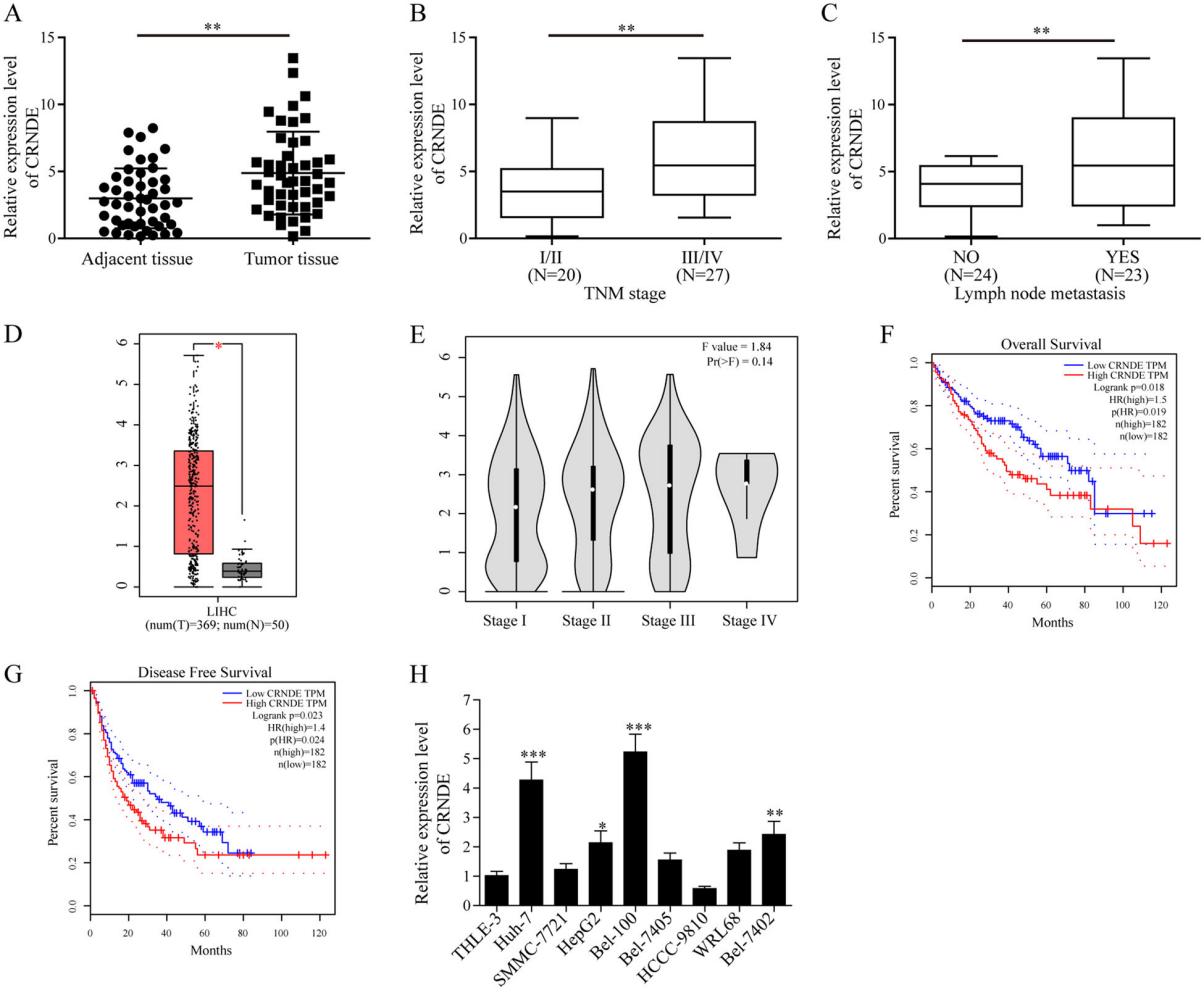
CRNDE was overexpressed in HCC and was correlated with clinical parameters. **a** Relative expression of CRNDE in HCC tumor tissues and adjacent tissues was determined by qPCR, *n* = 47. **b** Expression levels of CRNDE at different TNM stages were determined by qPCR. **c** Expression levels of CRNDE in tissue with lymph node metastasis or not were determined by qPCR. **d** A web server for analyzing the RNA sequencing expression data named GEPIA was accessed, and CRNDE expression in healthy human bodies and in cancer patients was compared in LIHC database. **e** GEPIA showed expression of CRNDE at TNM stages I, II, III, and IV in LIHC database. **f**, **g** Correlation between CRNDE expression and overall survival (**f**), or disease-free survival (**g**) were determined in GEPIA. (**h**) In vitro relative expression of CRNDE was compared in HCC cell lines HCCC-9810, the Bel-100, Bel-7402, Bel-7405, Huh-7, SMMC-7721, WRL68, HepG2, and the immortalized human hepatic cell line THLE3. *P < 0.05, **P < 0.01, and ***P < 0.001.

To investigate the potential biological functions of CRNDE in HCC development, we first examined its expression levels in HCC tumor tissues and the corresponding adjacent tissues (*n* = 47). In consistence with the previous findings^22,23,40^, we observed that CRNDE was upregulated in tumor samples from HCC patients (Fig. 1a). Next, patients were divided into two groups based on their TNM stages. We found that CRNDE exhibited higher expression at more advanced stages (III/IV) than at early stages (I/II; Fig. 1b). Furthermore, we analyzed the statistic correlation between CRNDE expression and lymph node metastasis. Interestingly, HCC cases with lymph node metastasis showed relatively high expression of CRNDE than those without lymph node metastasis (Fig. 1c).

To further substantiate our experimental observation above, we used GEPIA^41^, a web server for cancer and normal gene expression profiling and interactive analyses, and compared its expression in healthy human individuals (*n* = 50) and in HCC patients (*n* = 369). Indeed, the results from GEPIA database showed a higher expression of CRNDE in cancer patients than in heathy individuals (Fig. 1d). However, although CRNDE was differentially expressed at four TNM stages (I, II, III, and IV), no statistic changes were found between the four groups (Fig. 1e). Moreover, analysis of GEPIA database indicated that CRNDE expression was negatively correlated with both overall survival (Fig. 1f) and disease-free survival (Fig. 1g).

We next examined the in vitro expression of CRNDE. qPCR assays determined that CRNDE expression was remarkedly higher in the HCC cell lines (HCCC-9810, Bel-100, Bel-7405, Bel-7402, Huh-7, WRL68, SMMC-7721, and HepG2) than in the immortalized human hepatic cell line THLE3 cells (Fig. 1h). Collectively, these results confirmed that CRNDE was significantly upregulated in HCC patients and cell lines, and its expression profiles were statistically correlated with HCC clinical features.

### CRNDE knockdown inhibited HCC cell proliferation, migration and chemotherapy resistance

**Fig. 2:**
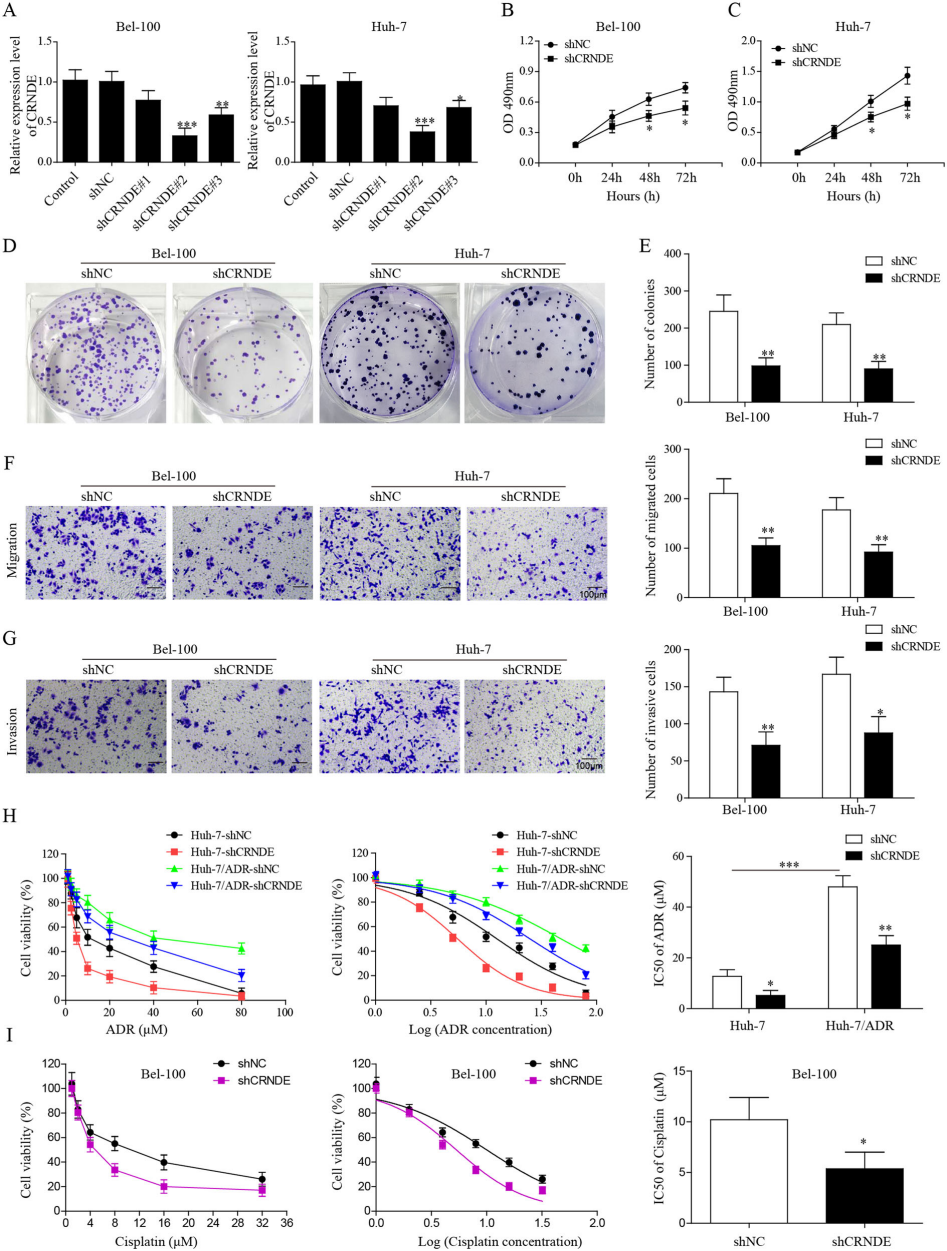
CRNDE knockdown inhibited HCC cell proliferation, migration, and chemotherapy resistance. The expression of CRNDE was constitutively knocked-down by transfecting Bel-100 and Huh-7 cells, with lentiviruses carrying CRNDE-specific shRNA (shCRNDE). Lentiviruses carrying negative control (shNC) were used as control. **a** Relative CRNDE expression was determined by qPCR. **b**, **c** Cell vitality was assessed by MTT assay in Bel-100 and Huh-7 cells transfected with shCRNDE or shNC. **d**, **e** Colony formation in Bel-100 and Huh-7 cells upon lentiviral infection was compared. **f**, **g** Transwell assay was performed to evaluate the cell migration (**f**) and invasion ability (**g**) after HCC cells were transfected with shCRNDE and shNC, respectively. **h** Huh-7 and Huh-7/ADR cells were transfected with shCRNDE or shNC, and relative cell viability was detected by MTT assay combined with a series concentration of ADR. IC_50_ of ADR was calculated in both Huh-7 and Huh-7/ADR cells. **i** were transfected with shCRNDE or shNC, and relative cell viability was detected by MTT assay combined with a series concentration of cisplatin. IC_50_ of cisplatin was analyzed in Bel-100 cells. The data were shown as mean ± SD based on at least three independent experiments. *P < 0.05, **P < 0.01, and ***P < 0.001.

To answer the question whether CRNDE upregulation was involved in HCC progression, we transfected Bel-100 and Huh-7 cells with lentiviruses encoding shCRNDE or shNC. The silencing effect was confirmed by qPCR assays (Fig. 2a). shRNA#2 was used for followed assays. As measured by MTT assay, viability of both Bel-100 and Huh-7 was largely reduced by shCRNDE transfection, as compared with shNC group (Fig. 2b, c). Cellular colony formation assay showed that much fewer colonies remained in Bel-100 and Huh-7 cells transfected with shCRNDE than in shNC group (Fig. 2d, e). We then performed transwell migration and invasion assays to assess whether CRNDE knockdown affected cell migration and invasive abilities. Our results confirmed that cell migration and invasive numbers were dramatically decreased in both HCC cell lines transfected with shCRNDE than in shNC group (Fig. 2f, g).

Chemoresistance is a serious clinical problem for HCC therapy and contributes largely to poor patient prognosis^7^. Therefore, we investigated whether ADR sensitivity in HepG2 and HepG2/ADR cell lines was affected by CRNDE knockdown. MTT assay results showed that both HepG2 and HepG2/ADR cells transfected with shCRNDE displayed reduced viability upon incubation, with the increasing concentrations of ADR for 24 h, as compared with cells transfected with empty viruses (Fig. 2h). A total of 50% inhibiting concentration value (IC_50_) of ADR significantly decreased in cells transfected with shCRNDE than in shNC group. (Fig. 2h). Moreover, silencing of CRNDE enhanced cisplatin sensitivity in BEL-100 cells and decreased significantly cisplatin IC_50_ value (Fig. 2i). All these results indicated that silencing of CRNDE efficiently suppressed HCC pathogenesis by inhibiting cell proliferation, colony formation, migration, and enhanced HCC cell chemosensitivity.

### Knockdown of CRNDE inhibited tumor growth and lung metastasis in xenograft model

**Fig. 3:**
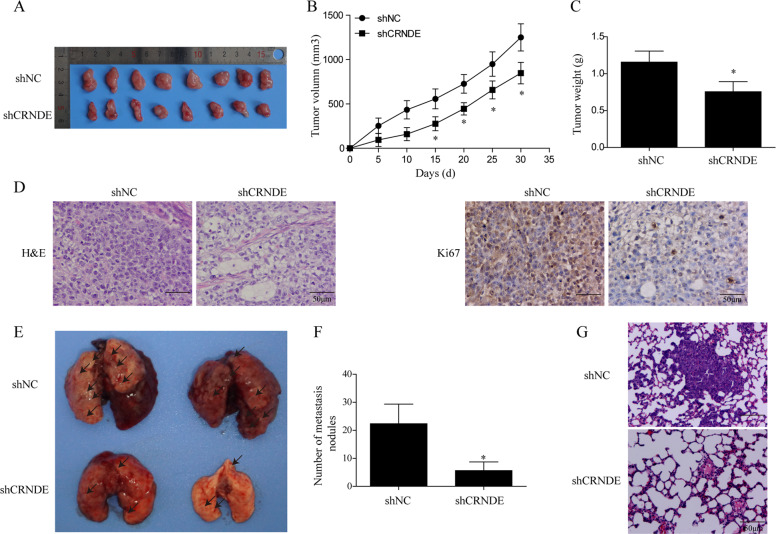
Knockdown of CRNDE inhibited tumor growth and lung metastasis in xenograft model. Xenograft tumor model was established by subcutaneously injecting the nude mice with Bel-100 cells transfected with lentiviruses carrying shCRNDE or shNC. **a** Mice were sacrificed 30 days after injection, tumors were collected and photographed. **b** Tumor sizes were measured every 5 days. **c** Tumor weight was compared between shCRNDE and shNC groups. **d** Tumor xenograft tissues were collected for H&E and IHC staining against Ki67, and representative images were shown. **e** Lungs were isolated from mice injected with shCRNDE or shNC into the tail veins and photographed. **f** Pulmonary metastasis nodules from two groups were counted. **g** Lung tissues were stained with H&E and images were taken under microscope. *N* = 5 for each group; *P < 0.05.

To determine whether HCC tumorigenesis could be affected by CRNDE in vivo, we next injected nude mice with Bel-100 cells that were transfected with lentiviruses carrying shCRNDE or shNC. We found that the volumes of the HCC xenograft tumors with knockdown of CRNDE were significantly smaller than those of shNC at 25 days after the HCC cell implantation (Fig. 3a). Tumor growth curve indicated that HCC tumor growth was effectively suppressed by CRNDE knockdown (Fig. 3b). In addition, tumor weight of the shCRNDE group (0.44 ± 0.07 g, *P* < 0.01) was markedly reduced as compared with the control group (0.21 ± 0.03 mm^3^, *P* < 0.01; Fig. 3c). The differences between xenograft tumors with CRNDE knockdown vs. those without were further confirmed by H&E and IHC staining for Ki67, a proliferated cell marker (Fig. 3d).

Because the lung is the most common site for the extrahepatic metastases and the prognosis for these patients are extremely poor^42^, we next determined the effects of CRNDE knockdown on pulmonary metastases from primary HCC. As shown in Fig. 3e, we found that lung metastases formed by CRNDE silencing group were significantly smaller than the shNC group. In addition, we counted the visible tumors on the lung surfaces and observed that the number of metastasis nodules formed in the CRNDE silencing group was largely reduced when compared to the control group (Fig. 3f). The differences of lung metastases between the two compared groups were further confirmed by H&E staining (Fig. 3g). Taken together, these findings demonstrated that in consistence with our in vitro experimental results, CRNDE silencing significantly inhibited HCC cell proliferation and extrahepatic migration in vivo.

### CRNDE was involved in epigenetic repression mediated by EZH2, SUZ12, and SUV39H1 on multiple tumor suppressor genes

**Fig. 4:**
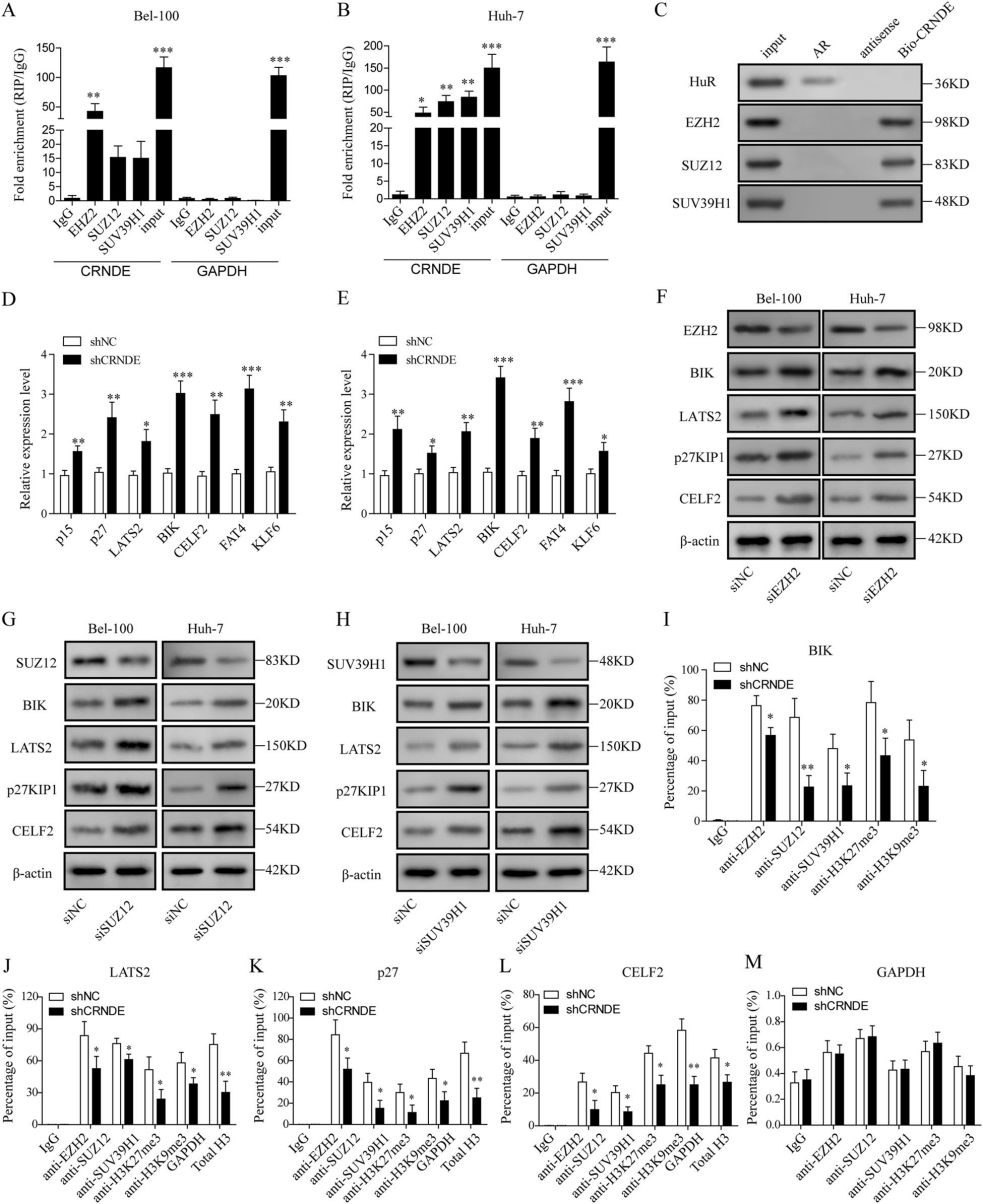
CRNDE was involved in epigenetic repression mediated by EZH2, SUZ12, and SUV39H1 on multiple tumor suppressor genes. qPCR was performed to measure CRNDE levels in immunoprecipitates in Bel-100 (**a**) and Huh-7 cells (**b**). CRNDE expression levels were displayed as fold changes relative to IgG immunoprecipitate. **c** RNA pull-down assay was applied to capture the proteins that bound CRNDE in Bel-100, and protein expression levels of HuR, EZH2, SUZ12, and SUV39H1 were determined by WB. HuR and AR were used as positive control. **d**, **e** qPCR analysis was performed to measure the relative expression levels of multiple tumor suppressor genes in Bel-100 (**d**) and Huh-7 cells (**e**). **f**, **g** HCC cells were transfected with lentiviruses encoding siRNAs against EZH2 (**f**), SUZ12 (**g**), SUV39H1 (**h**), or negative control and the protein expression levels of the silenced proteins together with BIK, LATS2, p27KIP1, and CELF2 were determined by WB. **i**–**l** HCC cells were transfected with shCRNDE or shNC, and ChIP-qPCR was used to identify the enrichment of SUZ12, SUV39H1, EZH2, H3K27me3, and H3K9me3 at promoter region of BIK (**i**), LATS2 (**j**), p27KIP1 (**k**), CELF2 (**l**), and GAPDH (**m**), respectively. The data were shown as Mean ± SD based on at least three independent experiments. *P < 0.05, **P < 0.01 and ***P < 0.001.

EZH2, SUZ12, and SUV39H1 have been found upregulated in a wide range of human cancer types, and enhanced cancer progression by repressing the transcription and expression of various tumor suppressor genes^43^. It was reported that CRNDE bound with EZH2 to facilitate tumor progression in colorectal cancer^18^. To understand the molecular mechanism underlying CRNDE upregulation in HCC cells, we employed RIP method to check the direct binding between CRNDE and EZH2, SUZ12, and SUV39H1. As presented in Fig. 4a, b, we detected the strong binding between CRNDE and EZH2, SUZ12, and SUV39H1 in both Bel-100 and Huh-7 cells. These results were further corroborated by RNA pull-down assay (Fig. 4c).

Next, we investigated the impact of CRNDE on several tumor suppressor genes that are reported to be inhibited by EZH2, SUZ12, and SUV39H1-mediated histone trimethylation. In this work, we measured the expression levels of p15, p27KIP1, LATS2, BIK, CELF2, FAT4, and KLF6 in Bel-100 and Huh-7 cells that were transfected with shCRNDE or shNC. As demonstrated in our qPCR and WB analysis, expression of these tumor suppressor genes was significantly upregulated when CRNDE was inhibited in both cell lines (Fig. 4d, e, Supplementary Fig. S1). To validate whether the expression of the tumor suppressor genes was affected by EZH2, SUZ12, and SUV39H1, we transfected Bel-100 and Huh-7 cells with siRNAs against EZH2, SUZ12, and SUV39H1. We observed that the selected protein levels, including BIK, LATS2, p27KIP1, and CELF2 were increased by inhibition of EZH2 (Fig. 4f), SUZ12 (Fig. 4g), or SUV39H1 (Fig. 4h). ChIP-qPCR approach was applied in this work to analyze whether the binding between SUZ12, SUV39H1, EZH2, H3K27me3, and H3K9me3 on tumor suppressor gene promoters was affected by CRNDE silencing. The results showed that the upon knockdown of CRNDE both cell lines displayed reduced enrichment of SUZ12, SUV39H1, EZH2, H3K27me3, H3K9me3 on BIK (Fig. 4i), LATS2 (Fig. 4j), p27KIP1 (Fig. 4k), and CELF2 (Fig. 4l). GAPDH showed no significant enrichment of indicated proteins (Fig. 4m). Protein analysis showed that knockdown of CRNDE did not changed the global level of H3K27me3 and H3K9me3 (Supplementary Fig. S2). Moreover, our qPCR analysis on RNA collected from ChIRP showed that BIK, LATS2, p27, and CELF2 promoters were retrieved by CRNDE-specific oligos, and CRNDE silencing markedly declined the retrieval rate (Supplementary Fig. S3). These results indicated the close interaction between CRNDE and EZH2/SUZ12/SUV39H1, which targeted directly the promoter loci of the tumor suppressor genes, and thus we reasoned that CRNDE might mediate epigenetic suppression of these proteins on multiple tumor suppressive genes by forming a complex with EZH2/SUZ12/SUV39H1, as well as the promoter regions of tumor suppressor genes.

### CELF2 inhibited HCC cell proliferation, migration, and chemoresistance and was involved in CRNDE-mediated oncogenic effect

**Fig. 5:**
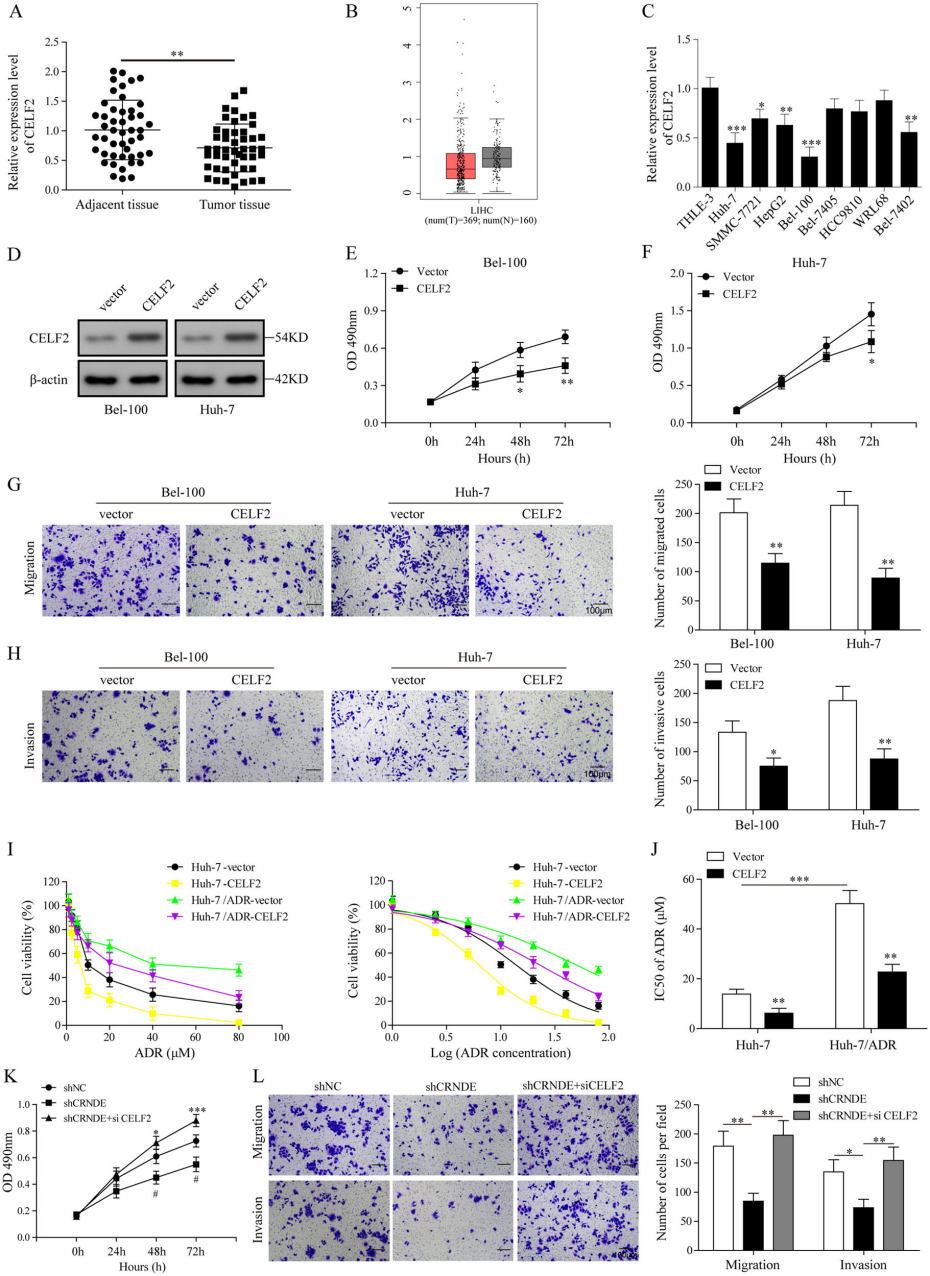
CELF2 inhibited HCC cell proliferation, migration, and chemoresistance and was involved in CRNDE-mediated oncogenic effect. **a** Relative expression of CELF2 in HCC tumor tissues and corresponding adjacent tissues was determined by qPCR (*n* = 47). **b** GEPIA was accessed and CELF2 expression in healthy human individual (*n* = 160) and in HCC patients (*n* = 369) was compared. **c** Expression of CELF2 was detected in HCCC-9810, Bel-100, Bel-7402, Bel-7405, HUH-7, SMMC-7721, HepG2, WRL68, and THLE3. **d** CELF2 was cloned into pcDNA3.1(+) and overexpressed in Bel-100 and Huh-7 cells. CELF2 expression was determined by WB and empty vectors were used as a negative control. **e**, **f** Cell vitality was evaluated by MTT assay in Bel-100 (**e**) and Huh-7 cells (**f**). **g**, **h** Cell migration and invasive abilities were determined by transwell assays in in Bel-100 (**g**) and Huh-7 cells (**h**). **i** Huh-7 and Huh-7/ADR cells were transfected with pcDNA3.1(+)-CELF2 or empty vectors, and relative cell viability was detected by MTT assay combined with ADR. **j** ADR IC_50_ was determined in both Huh-7 and Huh-7/ADR cells. **k** Bel-100 cell were transfected with shCRNDE alone or co-transfected with shCRNDE and siCELF2, cell viability was measured by MTT assay. **l** Transwell assay was used to determine the cell migration and invasive abilities in Bel-100 transfected with shCRNDE and/or siCELF2. The data were shown as mean ± SD based on at least three independent experiments. *P < 0.05, **P < 0.01, and ***P < 0.001.

Our results above indicated that CRNDE inhibited the expression of CELF2, an important tumor suppressor in several cancer types^44–46^. However, the implication of CELF2 in HCC has not yet been well understood to date. To answer the question whether CELF2 played a role in HCC pathogenesis, we detected the expression of CELF2 in human HCC tissues and adjacent tissues. As illustrated in our qPCR results, HCC tissues displayed decreased expression of CELF2 compared to the normal adjacent tissues (Fig. 5a). Next, we analyzed GEPIA-LIHC database, and compared the expression of CELF2 in healthy human bodies and in cancer patients (Fig. 5b). The results showed that expression of CELF2 in cancer patients (*n* = 369) was slightly lower than in heathy humans (*n* = 160). However, no statistical correlation between CRNDE and CELF2 was observed by analyzing our own databank (Supplementary Fig. S4a) and the TCGA database (Supplementary Fig. S4c). We further examined the relative expression of CELF2 in HCC cell lines. As observed in our qPCR results, expression of CELF2 was remarkedly lower in HCCC-9810, Bel-100, Bel-7405, Bel-7402, Huh-7, SMMC-7721, WRL68, and HepG2 than in THLE3 cells (Fig. 5c).

As described above, reduced expression was found in both HCC cancer patients and cell lines; therefore, we speculated that CELF2 may play important role in tumorigenesis and metastasis. To explore the function of CELF2 in HCC development, we transfected Bel-100 and Huh-7 cells with pcDNA3.1(+) vector encoding CELF2 or empty vectors, and validated the overexpression by WB (Fig. 5d). We then evaluated the effect of CELF2 on proliferation by using MTT assay. Remarkably, overexpression of CELF2 significantly decreased cell vitality in both cell lines (Fig. 5e, f). Inhibiting the effect of CELF2 on HCC cell tumorigenesis was further confirmed by colony formation assay and cells overexpressing CELF2 generated significantly less colonies (Supplementary Fig. S5a, b). Meanwhile, we speculated that CELF2 may have an impact on tumor metastasis, and measured the invasion and migration efficiency in HCC cells. As demonstrated in our transwell assays, overexpression of CELF2 blocked cell invasion and migration in both cell lines (Fig. 5g, h).

In this work, we also evaluated the impact of CELF2 overexpression on HCC drug resistance by treating the HepG2 and HepG2/ADR cells, with indicated concentration of ADR following CELF2 transfection. The MTT assay results indicated that cell viability of both cell lines decreased in an ADR-dose-dependent manner, and cells overexpressing CELF2 exhibited reduced viability than in those transfected with empty vectors (Fig. 5i). In accordance, IC_50_ of ADR was markedly lower in HCC cells transfected with CELF2 in comparison with control group (Fig. 5j). These results suggested that CELF2 functioned as an inhibitor in HCC cell proliferation, migration, and its overexpression enhanced ADR chemosensitivity. We were promoted by the findings above to explore whether inhibitive effect of CRNDE knockdown was reversed, when the cells were co-transfected with shCRNDE (shRNA target CRNDE) and siCELF2 (siRNA target CELF2). Interestingly, we found that inhibiting the effect of CRNDE silencing on HCC cell proliferation was totally abolished by CELF2 knockdown (Fig. 5k). In addition, cell invasion and migration abilities that were inhibited by shCRNDE were recovered upon co-transfection with siCELF2 (Fig. 5l). Taken together, all these data suggested a pivotal role of CELF2 in HCC tumorigenesis, migration, and chemoresistance, and we provided evidence that CELF2 silencing in HCC cell line 1 reversed the inhibiting effect of shCRNDE on cell viability, invasion, and migration.

### LATS2 compromised the promoting effect of CRNDE on HCC cell proliferation, migration, chemoresistance, and its inhibition on the Hippo pathway

**Fig. 6:**
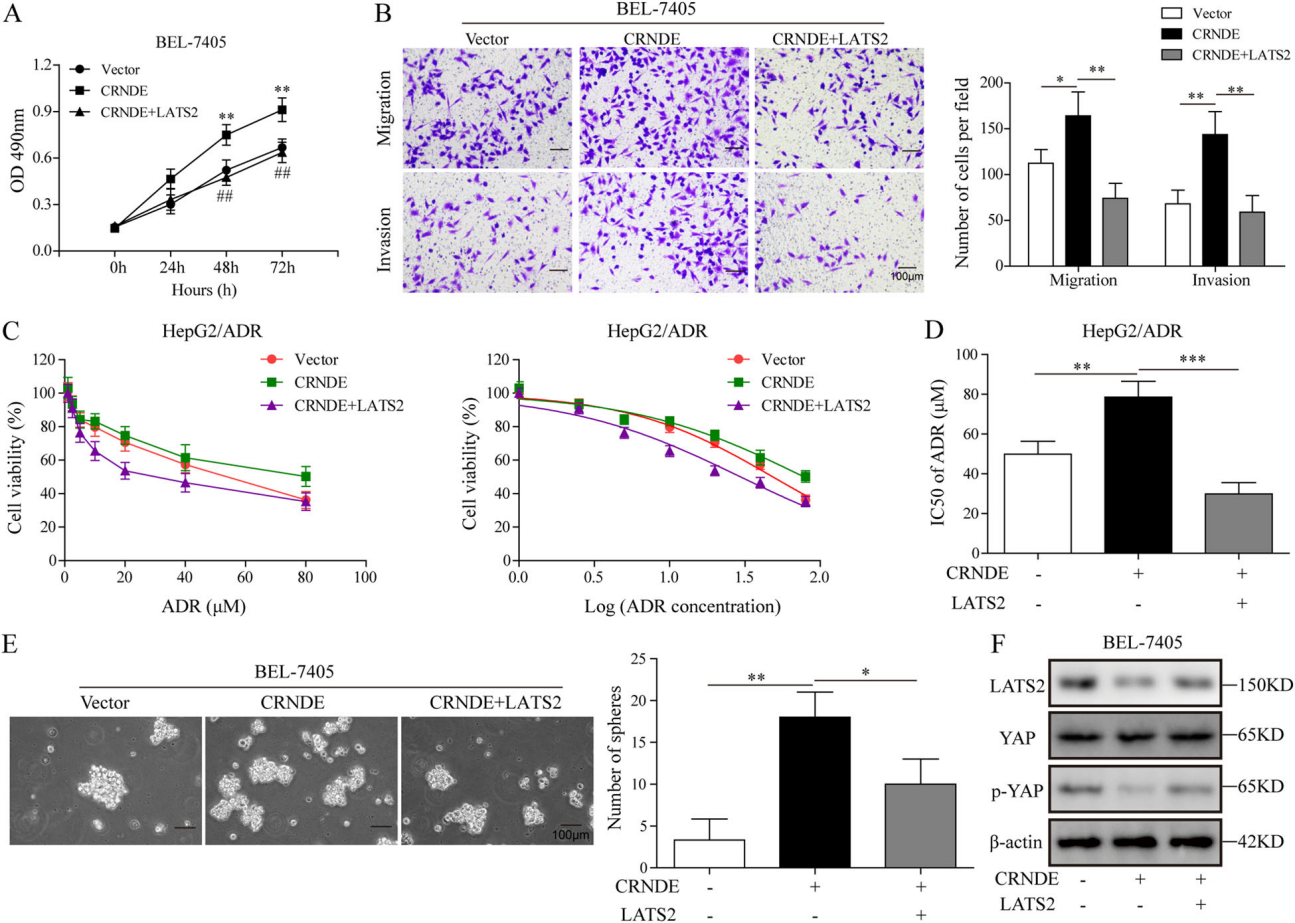
LATS2 compromised the promoting effect of CRNDE on HCC cell proliferation, migration, chemoresistance, and its inhibition on the Hippo pathway. Bel-7405 cells were transfected with pcDNA3.1(+) vectors containing CRNDE and/or pcDNA3.1(+) vectors containing LATS2. **a** MTT assay was carried out to measure the cell viability. **b** Cell migration and invasive abilities were determined by transwell assay. **c** Huh-7/ADR cells were transfected with lentiviruses encoding CRNDE and/or LATS2, and cell viability was measured by MTT assay when ADR was added. Cells transfected with empty vectors were used as negative control. **d** ADR IC_50_ was determined in Huh-7/ADR cells overexpressing CRNDE and/or LATS2. **e** Sphere-forming capacity was assessed and shown. **f** WB was used to evaluate expression levels of Hippo pathway components in Bel-7405 cells overexpressing CRNDE and/or LATS2. The data were shown as mean ± SD based on at least three independent experiments. *P < 0.05, **P < 0.01, and ***P < 0.001.

Our results above showed that expression of LATS2 was significantly inhibited by CRNDE. The Hippo signaling pathway has been recently established as a key regulator in human cancers, and many of the pathway elements function as tumor suppressors^47,48^. LATS2, (large tumor suppressor kinase 2, also known as KPM) plays a central role in mediating the Hippo signaling pathway, and the dysregulated LATS2 expression has been reported to be involved in HCC tumorigenesis^49^. Thus, in the present work, we reasoned that that the regulation of CRNDE in HCC was mediated by LATS2. To test this hypothesis, we transfected Bel-7405 cells, which displayed relative low expression of CRNDE, with CRNDE and LATS2 expressing plasmids simultaneously, to investigate whether overexpression of CRNDE could induce malignant phenotypes. As demonstrated in our MTT assay results, LATS2 overexpression compromised the promoting effect of CRNDE on HCC cell viability (Fig. 6a). Intriguingly, transwell assay indicated that cellular invasion and migration abilities were significantly lower in HCC-9810 cells co-transfected with CRNDE and LATS2 in comparison with those transfected with CRNDE alone (Fig. 6b). Next, we aimed to test whether ADR chemosensitivity was altered when CRNDE and LATS2 were co-transfected. Indeed, HepG2/ADR cells that were co-transfected with CRNDE and LATS2 displayed reduced chemoresistance in comparison to those transfected with CRNDE alone or our negative control, as demonstrated in our MTT assay results (Fig. 6c). IC_50_ of ADR was significantly reduced and chemoresistance changes observed above were further confirmed (Fig. 6d). In addition, HCC-9810 cells co-transfected with CRNDE and LATS2 formed remarkably less spheres than those transfected with CRNDE (Fig. 6e). YAP1 is another Hippo pathway effector and previous study demonstrated that LATS2 inhibited tumorigenesis of HCC cells by upregulating YAP1 phosphorylation^49^. As indicated in our WB results, CRNDE inhibited the expression of LATS2, p-YAP but not YAP, and the repressive effect was abolished by co-expression of LATS2 (Fig. 6f). Consistently, we found that CRNDE silencing suppressed the mRNA expression of YAP target genes cysteine-rich 61 (CYR61) and connective tissue growth factor, which were reported to be upregulated in HCC specimens^50^ (Supplementary Fig. S6). These results demonstrated that CRNDE overexpression was able to facilitate HCC cell line acquire malignant phenotype and LATS2 overexpression counteracted the enhancing effect of CRNDE on proliferation, migration, tumorigenesis, as well as its inhibition on the Hippo pathway.

## Discussion

LncRNAs have emerged recently as promising players in gene expression regulation through novel mechanisms that are distinguished from mRNA-encoding genes. Dysregulated lncRNAs were implicated in a broad range of disease including cancers. In consistence with previous reports, we confirmed in the present study that the lncRNA CRNDE was markedly upregulated in HCC patients, and cell lines as compared with the healthy tissues or non-HCC cell lines. The overexpression of CRNDE was statistically correlated with dismal patient prognosis and poor clinical outcomes, such as advanced TNM stages, positive lymph node metastasis, and reduced survival rate. Furthermore, we showed that CRNDE targeted directly at EZH2, SUZ12, and SUV39H1 in HCC and promoted HCC progression by mediating their inhibition on various tumor suppressor genes, including CELF2 and LATS2.

CRNDE has been reported in previous studies to act as an oncogene in diverse human cancer types^18–21^. For instance, Chen et al., Jiang et al., and Tang et al. demonstrated that CRNDE promoted HCC cell proliferation, migration, and invasion through distinct molecules or signal pathways^22,23^. We observed similar effects in our present study, and further explained the molecular mechanisms underlying CRNDE-regulated HCC progression. PRC2 is recognized as important and context-dependent tumor suppressive or oncogenic molecule, and its subunits EZH2 and SUZ12 were reported to inhibit the the expression of numerous tumor suppressor genes by influencing H3K9me and H3K27me3 levels^29–32^. Intriguingly, multiple nuclear lncRNAs were found to interact with PRC2 and collaboratively regulated the epigenetic status in cancer cells^36,37^. Specifically, lncRNA CRNDE was reported as an oncogene in colorectal cancer by associating with EZH2 (ref. ^18^). In the present study, we confirmed the binding between CRNDE and EZH2 in HCC. In addition, we provided the first evidence that CRNDE interacted with SUZ12 and SUV39H1 via direct or indirect binding, and effectively modulated their inhibition on tumor suppressor gene in HCC progression.

CELF2, also known as CUGBP2, is an RNA-binding protein and was reported to be implicated in several human cancers by acting as splicing regulator in nucleus^44,51^. Although CELF2 has received greater attention in recent years, its functions haven’t been broadly studied^52,53^. Our investigations demonstrated for the first time that CELF2 was markedly downregulated in HCC patients and cell lines, and it functioned as a tumor suppressor gene in HCC. Nevertheless, further research is required to elucidate the exact mechanisms underlying the CELF2-mediated HCC inhibition and chemosensitivity.

Drug resistance arises commonly in cancers and leads to the failure of chemotherapy in nearly all advanced patients^54,55^. HCC is one of the most malignant cancer types yet displayed rather low chemosensitivity^56,57^. In order to finally overcome the chemoresistance and maximize the chemotherapy benefits in HCC, many efforts should be made toward a better understanding of the underlying mechanism^58^. Growing evidence showed that lncRNAs played crucial roles in HCC chemoresistance regulation^59^. Our findings indicated that the lncRNA CRNDE influenced the HCC drug resistance via p27KIP1, BIK, LATS2, and CELF2, which were proven to be involved in chemosensitivity of cancel cells^60–62^. For instance, p27KIP1 and BIK have been extensively studied, and were reported to modulate the chemosensitivity in malignant glioma, cholangiocarcinoma, and breast cancer^63–66^. It has been also shown that LATS2, the core kinase of Hippo pathway, functions as tumor suppressor gene in various cancers types, including leukemia^67^, breast cancer^68^, lung cancer^69^, and prostate cancer^70^ through the regulated phosphorylation of the transcription co-activators YAP and TAZ (transcriptional co-activator with PDZ domain)^71,72^. In addition, the loss of LATS2 has been demonstrated to be associated with increased chemotherapeutic drug resistance in prostate cancer^73^ and leukemia^62^. Our research revealed that CRNDE-regulated chemosensitivity of HCC cell line by influencing LATS2 and Hippo signal pathway, which might be a potential mechanism by that CRNDE mediated chemoresistance in HCC. However, the lack of correlation between CRNDE and CELF2 or LATS2 suggested that CELF2 and LATS2 may not be regulated by CRNDE alone. And these negative results might limit our finding apply in clinical research and remind us we should draw our conclusion more carefully.

In conclusion, our data provide the evidence that CRNDE play important roles in HCC that is related to mediate epigenetic suppression of multiple tumor suppressive genes, especially CELF2 and LATS2.

## Supplementary information

figureS1

figureS2

figureS3

figureS4

figureS5

supplemental figure legends

figureS6
