# Supplementary figures and images for: LncRNA CRNDE facilitates epigenetic suppression of CELF2 and LATS2 to promote proliferation, migration and chemoresistance in hepatocellular carcinoma
Source: Cell Death Dis. 2020 Aug 11;11(8):676. doi: 10.1038/s41419-020-02853-8 (PMC7442829; doi:10.1038/s41419-020-02853-8)

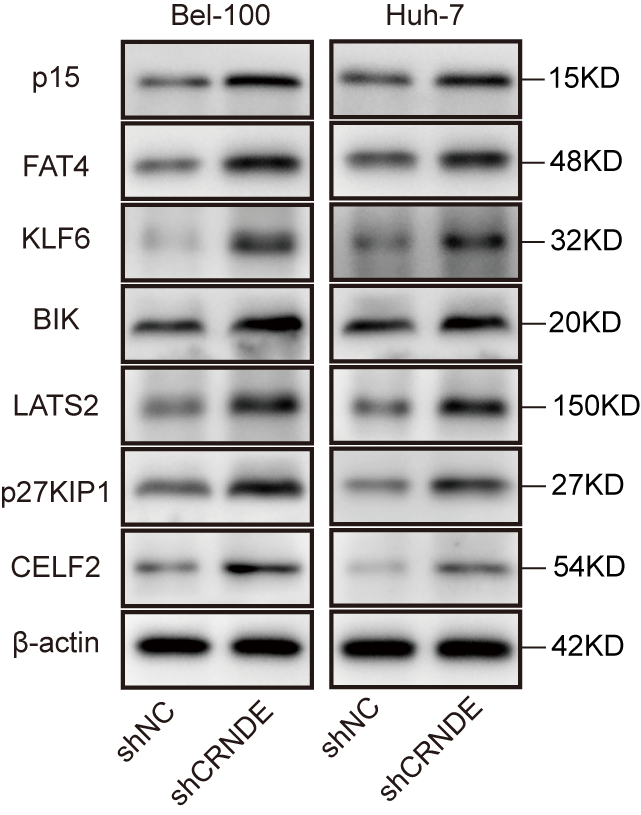

Supplement: Supplementary file 1 — figureS1 [file 41419_2020_2853_MOESM1_ESM.tif]

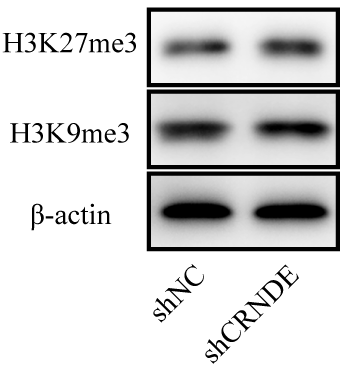

Supplement: Supplementary file 2 — figureS2 [file 41419_2020_2853_MOESM2_ESM.tif]

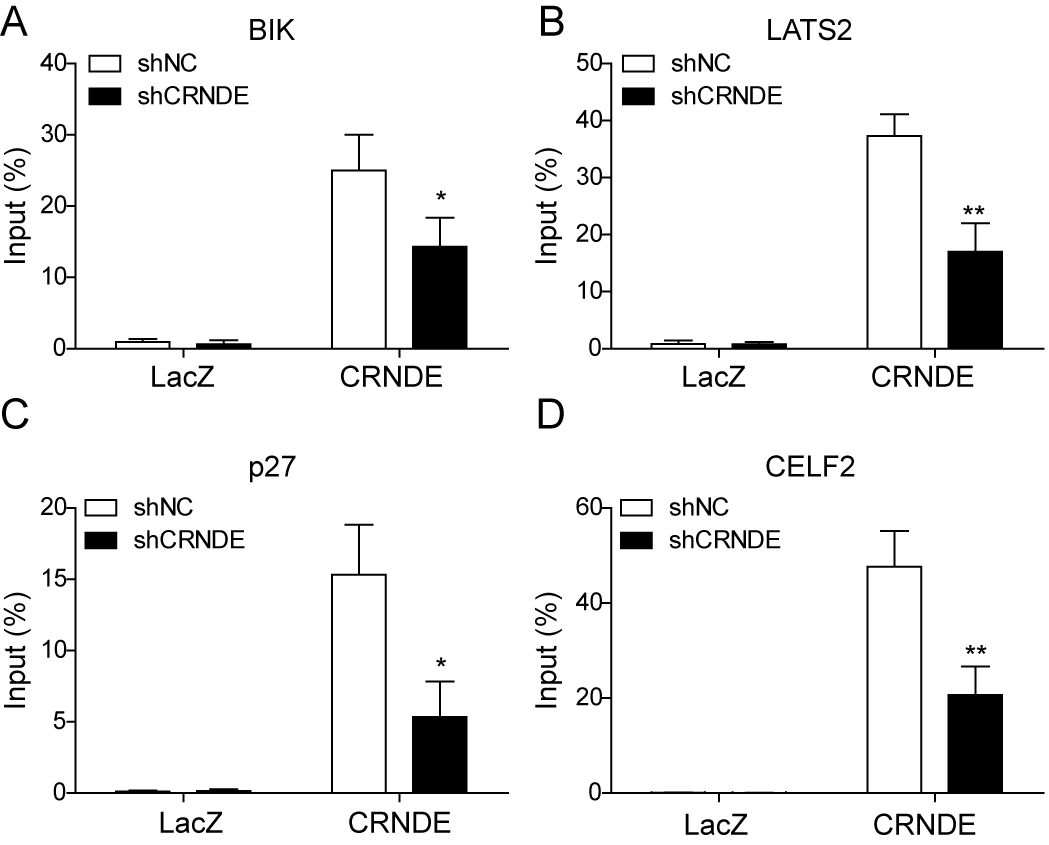

Supplement: Supplementary file 3 — figureS3 [file 41419_2020_2853_MOESM3_ESM.tif]

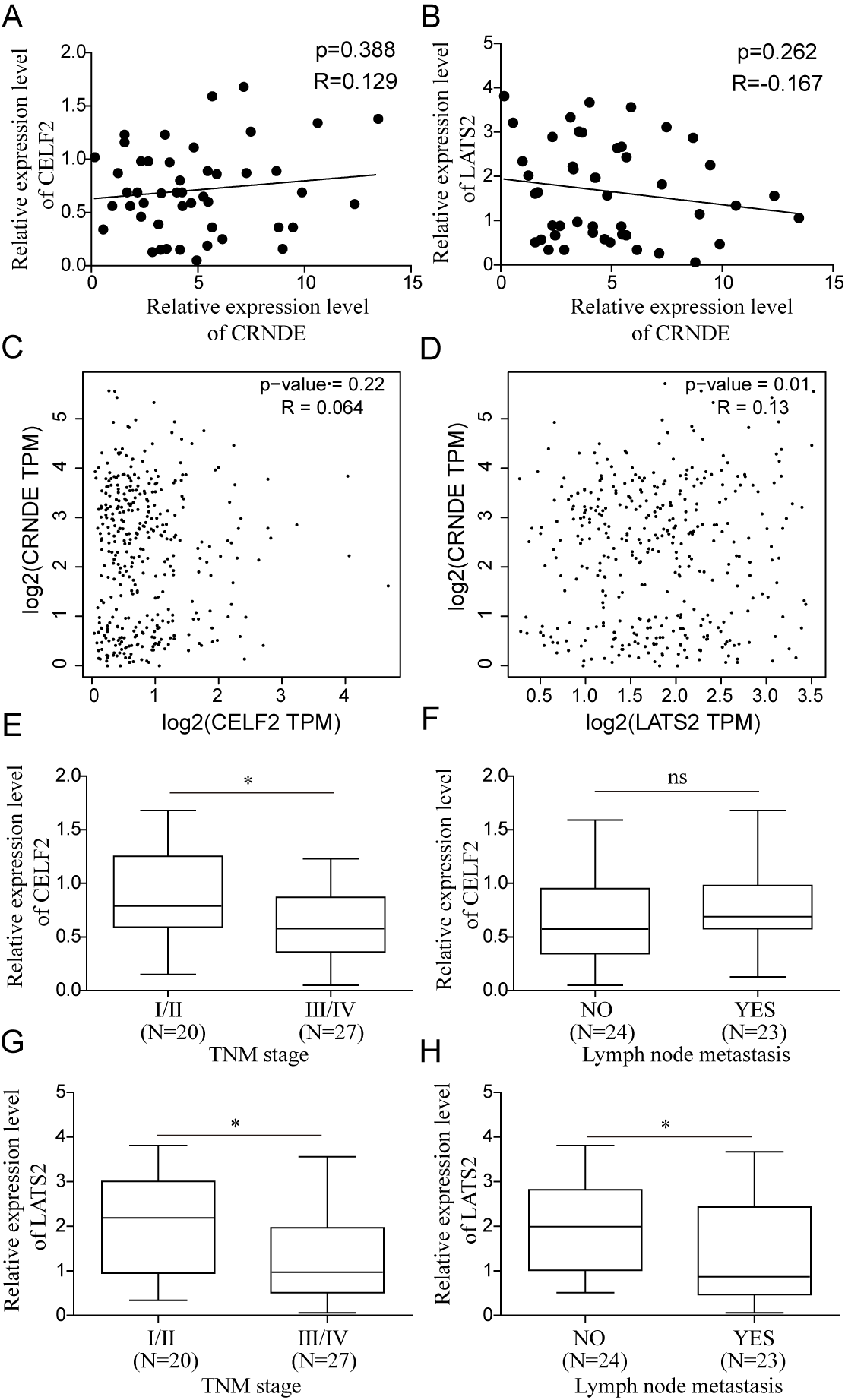

Supplement: Supplementary file 4 — figureS4 [file 41419_2020_2853_MOESM4_ESM.tif]

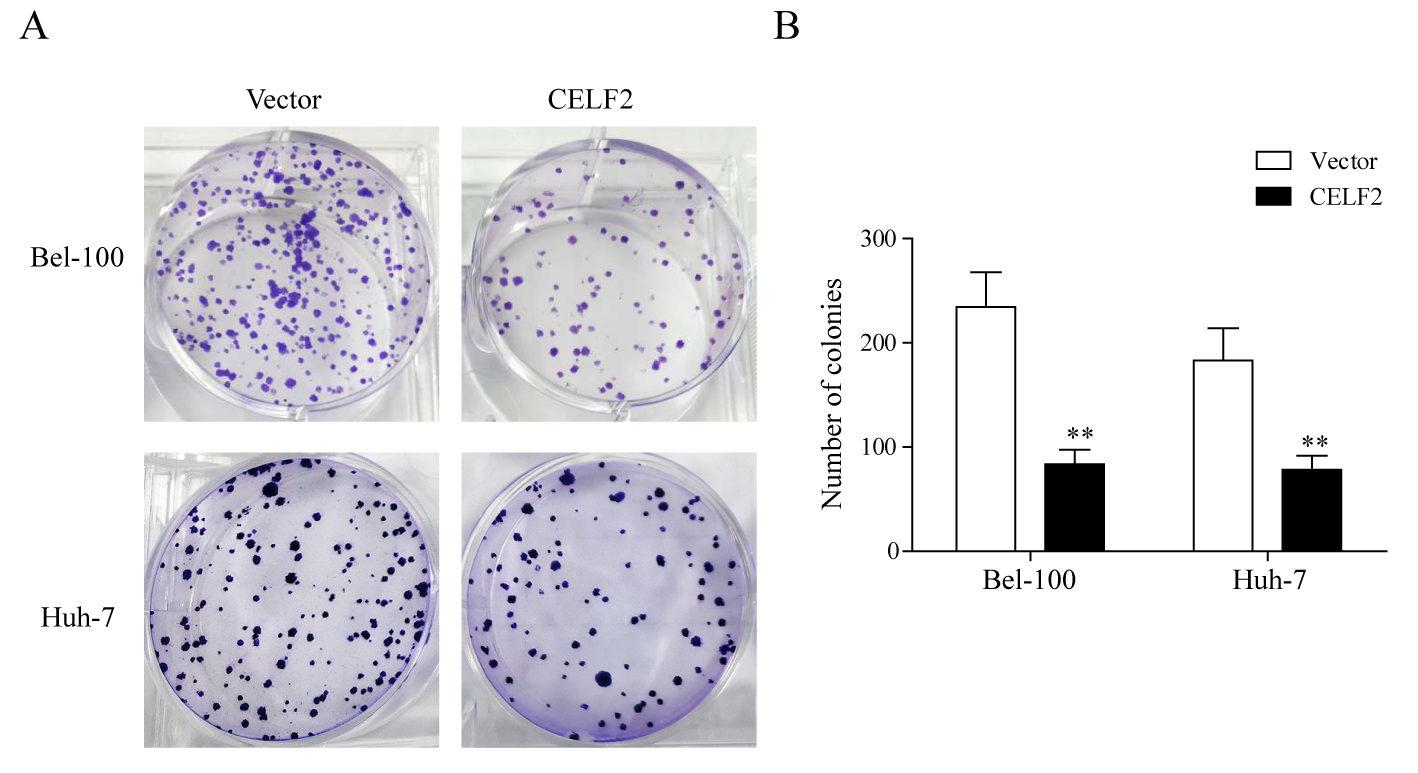

Supplement: Supplementary file 5 — figureS5 [file 41419_2020_2853_MOESM5_ESM.tif]

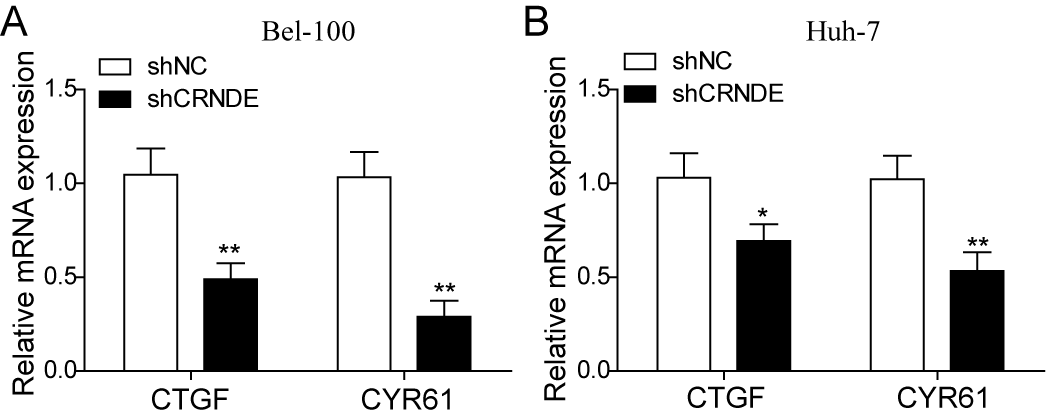

Supplement: Supplementary file 7 — figureS6 [file 41419_2020_2853_MOESM7_ESM.tif]
