# Supplementary material for: LncRNA CRNDE facilitates epigenetic suppression of CELF2 and LATS2 to promote proliferation, migration and chemoresistance in hepatocellular carcinoma
Source: Cell Death Dis. 2020 Aug 11;11(8):676. doi: 10.1038/s41419-020-02853-8 (PMC7442829; doi:10.1038/s41419-020-02853-8)
Supplement: Supplementary file 6 — supplemental figure legends [file 41419_2020_2853_MOESM6_ESM.docx]

**Figure S1. WB analysis was conducted to compare the protein expression of p15, p27, LATS2, BIK, CELF2, FAT4 and KLF6 in Bel-100 and Huh-7 cells transfected with shCRNDE or shNC.**

**Figure S2. Protein level of H3K27me3 and H3K9me3 in BEL-100 cells which were transfected with shCRNDE or shNC were determined by western blotting.**

**Figure S3. ChIRP experiment was conducted to detect the interaction between CRNDE and promoter regions of the tumor suppressor genes** (A) BIK, (B) LATS2, (C) p27 and (D) CELF2. LacZ was used as negative control.

**Figure S4. Correlation analysis of CELF2 or LATS2 with CRNDE, TNMG stage and lymphatic metastasis.** Pearson analysis of the statistical correlation between CRNDE and CELF2 (A) as well as LATS2 (B). Correlation between CRNDE expression in TCGA database and CELF2 (C) as well as LATS2 (D) was analyzed by using GEPIA web server. (E) Expression levels of CELF2 at different TNM stages were determined by qPCR. (F) Expression levels of CRNDE in tissue with lymph node metastasis or not were determined by qPCR. (G) Expression levels of LATS2 at different TNM stages were determined by qPCR. (H) Expression levels of LATS2 in tissue with lymph node metastasis or not were determined by qPCR.

**Figure S5. CELF2 reduced colony formation in Bel-100 and Huh-7 cells.** (A) Bel-100 and Huh-7 cells were transfected with CELF2 or empty vectors and colony formation assay was performed. (B) Colonies that were formed were counted. The data were shown as Mean ±SD based on at least three independent experiments. **P<0.01.

**Figure S6. Relative mRNA expression levels of two YAP target genes CYR61 and CTGF in BEL-100.** (A) and Huh-7 cells (B) which were transfected with shCRNDE or shNC were determined by qPCR analysis.
